# Color-tunable properties of Eu^3+^- and Dy^3+^-codoped Y_2_O_3_ phosphor particles

**DOI:** 10.1186/1556-276X-7-556

**Published:** 2012-10-08

**Authors:** Timur Sh. Atabaev, Yoon-Hwae Hwang, Hyung-Kook Kim

**Affiliations:** 1 Department of Physics and Astronomy, Seoul National University, Seoul, 151-747, Republic of Korea; 2 Department of Nanomaterials Engineering and BK21 Nano Fusion Technology Division, Pusan National University, Miryang, 627-706, Republic of Korea

**Keywords:** Y_2_O_3_ particles, Luminescence, Urea homogeneous precipitation, Eu^3+^ and Dy^3+^ codoped

## Abstract

Rare-earth phosphors are commonly used in display panels, security printing, and fluorescent lamps, and have potential applications in lasers and bioimaging. In the present study, Eu^3+^- and Dy^3+^-codoped uniform-shaped Y_2_O_3_ submicron particles were prepared using the urea homogeneous precipitation method. The structure and morphology of the resulting particles were characterized by X-ray diffraction, field emission scanning electron microscope, and field emission transmission electron microscope, whereas their optical properties were monitored by photoluminescence spectroscopy. The room-temperature luminescence color emission of the synthesized particles can be tuned from red to yellow by switching the excitation wavelength from 254 to 350 nm. The luminescence intensities of red and yellow emissions could be altered by varying the dopant concentration. Strong quenching was observed at high Eu^3+^ and Dy^3+^ concentrations in the Y_2_O_3_ host lattice.

## Background

The development of novel luminescent phosphor materials with a controllable size and morphology has been a major focus in the field of photonics and optoelectronics [1]. Phosphor nanocrystals are exceptionally promising materials in many fields of technology including photonics, luminescent displays, fluorescent lamps, lasers, cathodoluminescence, and biotechnology [2]. Moreover, the emission wavelength of rare-earth-doped nanoparticles is independent of the particle size and depends only on the dopant type, leading to lower synthesis cost. They also offer excellent chemical stability as well as high quantum yield. Different methods have been used to fabricate nanocrystalline phosphor particles, such as flame spray pyrolysis [3], co-precipitation method [4], sol–gel method [5], and solvothermal method [5]. The urea homogeneous precipitation method was recognized to be a green route for the high-yield mass production of spherical ceramic submicron particles with controllable sizes. Spherical-shaped particles can improve the optical performance due to the high packing density and reduction of light scattering [6].

Yttrium oxide (Y_2_O_3_) has been investigated widely as a host material for rare-earth (RE) ion doping in optical applications [3,4,6,7] on account of its excellent chemical stability, broad transparency range (0.2 to 8 μm) with a band gap of 5.6 eV, high refractive index, and low phonon energy [1]. Furthermore, the similarities in the chemical properties and ionic radius of RE ions and Y_2_O_3_ make it an attractive choice as a host material [6,8].

The color tunability of yttria-based phosphors can be achieved by codoping the host material with some specific rare-earth elements. For example, in our previous report we investigated the color-tunability effect of Eu^3+^- and Tb^3+^-codoped Y_2_O_3_ submicron particles [8]. We showed that the color emission of synthesized particles could be tuned precisely from red to green by a simple variation of the Tb/Eu ratio and excitation wavelength. Strong energy transfer (ET) from Tb to Eu ions was observed in Tb/Eu-codoped Y_2_O_3_ submicron particles, but back ET from Eu^3+^ to Tb^3+^ was not significant. Ishiwada et al. investigated the Tb/Tm-codoped Y_2_O_3_ phosphor for high-temperature thermometry application [9]. The synthesized Tb/Tm-codoped Y_2_O_3_ phosphor showed a distinct change of visible emission colors from green to blue with increasing temperature. Therefore, research into Y_2_O_3_ codoped with other different RE activators is important because the color-tunable properties can be used in a wide range of applications. Although many studies have examined the optical properties of RE ion-doped Y_2_O_3_ phosphors, only a few have investigated the codoping of two or more different ions in the same yttria host material [6,8,9].

In recent years, the Eu/Dy codoping in a single host material has attracted a great deal of attention and has been extensively investigated. For example, the SrAl_2_O_4_ host material codoped with Eu^3+^ and Dy^3+^ is known as a new generation long-lasting luminescent phosphor material [10]. It is well known that Eu^3+^ doping into Y_2_O_3_ host material results in a red emission, whereas doping with Dy^3+^ results in blue-yellow emission [11]. To the best of the authors' knowledge, there are no reports of an Y_2_O_3_ phosphor codoped with Eu^3+^ and Dy^3+^. In addition, there is an optimum concentration of dopant ions for all RE phosphors, but the optimum concentration also depends on several parameters, such as the size of the phosphors and synthetic route.

In the present study, urea homogeneous precipitation synthesis method was used for the preparation of Eu^3+^- and Dy^3+^-codoped Y_2_O_3_ submicron particles. The morphology and particles characteristics were investigated by X-ray diffraction (XRD), field emission scanning electron microscopy (FESEM), field emission transmission electron microscopy (FETEM), and energy dispersive X-ray (EDX) spectroscopy. The optical properties of the synthesized particles were explored by photoluminescence spectroscopy (PL). Y_2_O_3_ submicron particles with different concentrations of codoped Eu^3+^ and Dy^3+^ were investigated, and the luminescence intensity of these particles were found to be strongly dependent on the activators concentration. The emission color of the Eu^3+^- and Dy^3+^-codoped Y_2_O_3_ particles could be switched from red to yellow by variation of the excitation wavelength.

## Methods

### Chemical synthesis

Analytical grade Y_2_O_3_ (99.9%), europium oxide (Eu_2_O_3_; 99.9%), dysprosium oxide (Dy_2_O_3_; 99.9%), nitric acid (HNO_3_; 70%), and urea (99% to 100.5%) were purchased from Sigma-Aldrich Corporation (MO, USA) and were used without further purification.

Uniform-shaped sub-micron Eu^3+^- and Dy^3+^-codoped Y_2_O_3_ particles were synthesized according to the reported protocols [6,8]. Phosphor precipitates were prepared by heating the corresponding RE nitrates (0.001 mol each sample) in aqueous solution of urea (40 ml H_2_O and 0.5 g urea). The concentration of Eu^3+^ varied between 1 to 3 mol%, whereas the Dy^3+^ concentration varied from 1 to 2 mol%.

### Physical characterization

The structure of the prepared powders was examined by XRD using a Bruker D8 Discover diffractometer (Bruker Optics Inc., MA, USA) with Cu Kα radiation (*λ* = 0.15405 nm) and a 2*θ* scan range of 20 to 60°. The structural properties were also analyzed using Fourier transform infrared spectroscopy (Jasco FT/IR6300, JASCO Corp., Easton, MD, USA). The morphologies of the particles were characterized by FESEM (Hitachi S-4700, Hitachi, Ltd., Tokyo, Japan) and FETEM (JEOL JEM-2100F, JEOL Ltd., Tokyo, Japan). Elemental analysis was carried out by EDX (Horiba 6853-H, HORIBA Jobin Yvon Inc., Edison, NJ, USA). The PL measurements were performed with a Hitachi F-7000 spectrophotometer equipped with a 150-W xenon lamp as an excitation source. All the measurements were performed at room temperature.

## Results and discussion

### Morphology and structure

**Figure 1:**
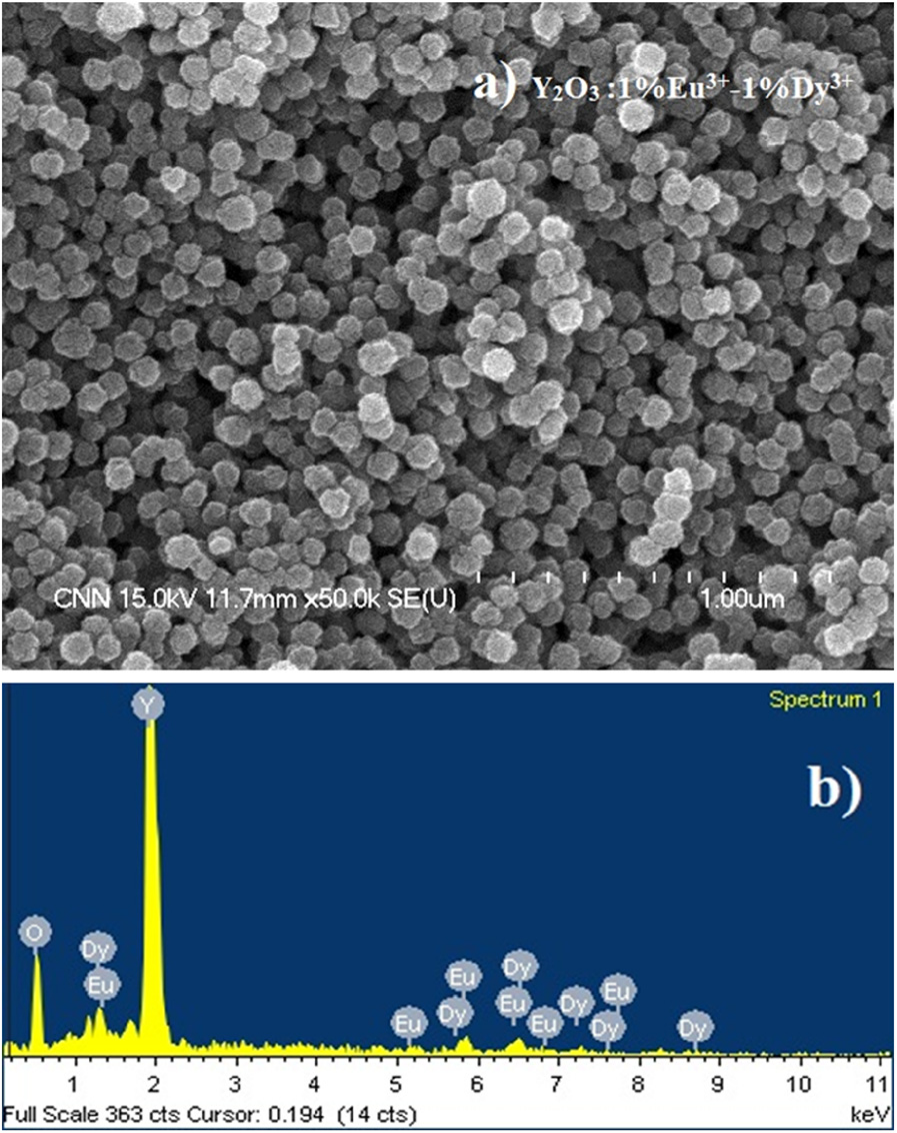
**Y**_**2**_**O**_**3**_**: 1%Eu**^**3+**^**-1%Dy**^**3+**^**phosphor particles (*T***_**cal.**_**= 1,000°C).** FESEM image (**a**) and EDX spectra (**b**).

The luminescence intensity depends strongly on the phosphor crystallinity [6,11]. Therefore, all synthesized particles were calcinated at the temperature of 1,000°C. The morphology of the synthesized phosphor particles after calcination at 1,000°C was examined by FESEM. Figure 1a shows FESEM image of the Y_2_O_3_ phosphor particles codoped with a 1%Eu^3+^-1%Dy^3+^ composition. From the FESEM image, it is apparent that the Y_2_O_3_:1%Eu^3+^-1%Dy^3+^ phosphor particles consists of relatively uniform, spherical-shaped submicron particles, 100 ± 20 nm in size. 

**Figure 2:**
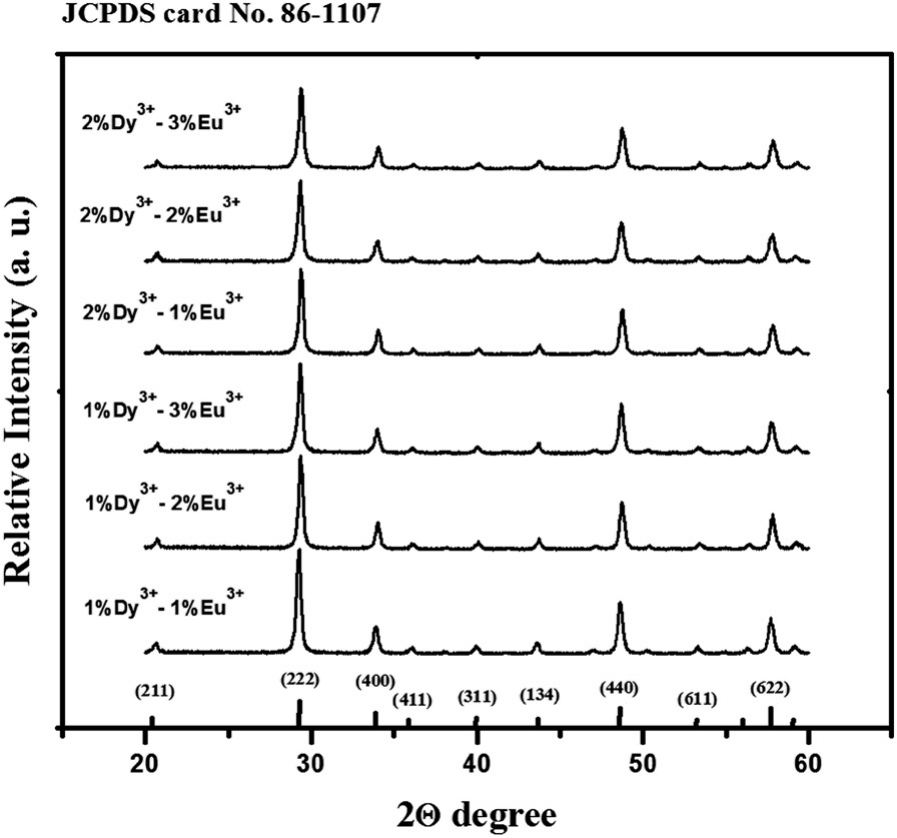
**X-ray diffraction patterns of Y**_**2**_**O**_**3**_**phosphor particles.** The Y_2_O_3_ particles are codoped with different RE ion (Eu^3+^ and Dy^3+^) concentrations (*T*_cal._ = 1,000°C).

Codoping with different Eu^3+^ and Dy^3+^ concentrations did not alter the morphology of the final phosphor product, and all particles had a spherical morphology with a size distribution of 100 ± 20 nm (not shown for other samples). On the other hand, we showed that the sizes of the final Y_2_O_3_ phosphor particles can be tuned by altering the reaction time, reaction temperature, and concentration of starting materials [8]. The EDX analysis of 1%Eu^3+^-1%Dy^3+^-codoped Y_2_O_3_ particles confirmed the presence of dysprosium and europium elements in the yttria host material (Figure 1b). Figure 2 shows the XRD patterns of Y_2_O_3_ submicron particles codoped with different Eu^3+^ and Dy^3+^ compositions, and the standard peak positions of a pure cubic Y_2_O_3_ structure (JCPDS No. 86–1107). 

It is obvious that all the diffraction peaks could be indexed directly to the cubic Y_2_O_3_ phase with the space group *Ia3* (206) according to the standard card (JCPDS No. 86–1107). No additional peaks from the doped components could be detected due to a relatively low concentration of dopant ions indicating the formation of a pure cubic Y_2_O_3_ phase. Table 1 lists the mean crystallite sizes of synthesized particles estimated from the well-known Debye-Scherrer's equation. The diffraction data of the three strongest peaks ({222}, {440}, and {622} planes) were used to calculate the mean crystallite sizes. The crystallite sizes showed a slightly increasing tendency, which can be attributed to the effect of the increased dopant concentration [8]. 

**Table 1:** **Calculated mean crystallite sizes of Eu**^**3+**^- **and Dy**^**3+**^-**codoped Y**_**2**_**O**_**3**_**particles**

**1Dy/1Eu**	**1Dy/2Eu**	**1Dy/3Eu**	**2Dy/1Eu**	**2Dy/2Eu**	**2Dy/3Eu**
36.42 ± 0.11	36.72 ± 0.09	36.96 ± 0.10	36.67 ± 0.14	37.01 ± 0.16	37.28 ± 0.07

**Figure 3:**
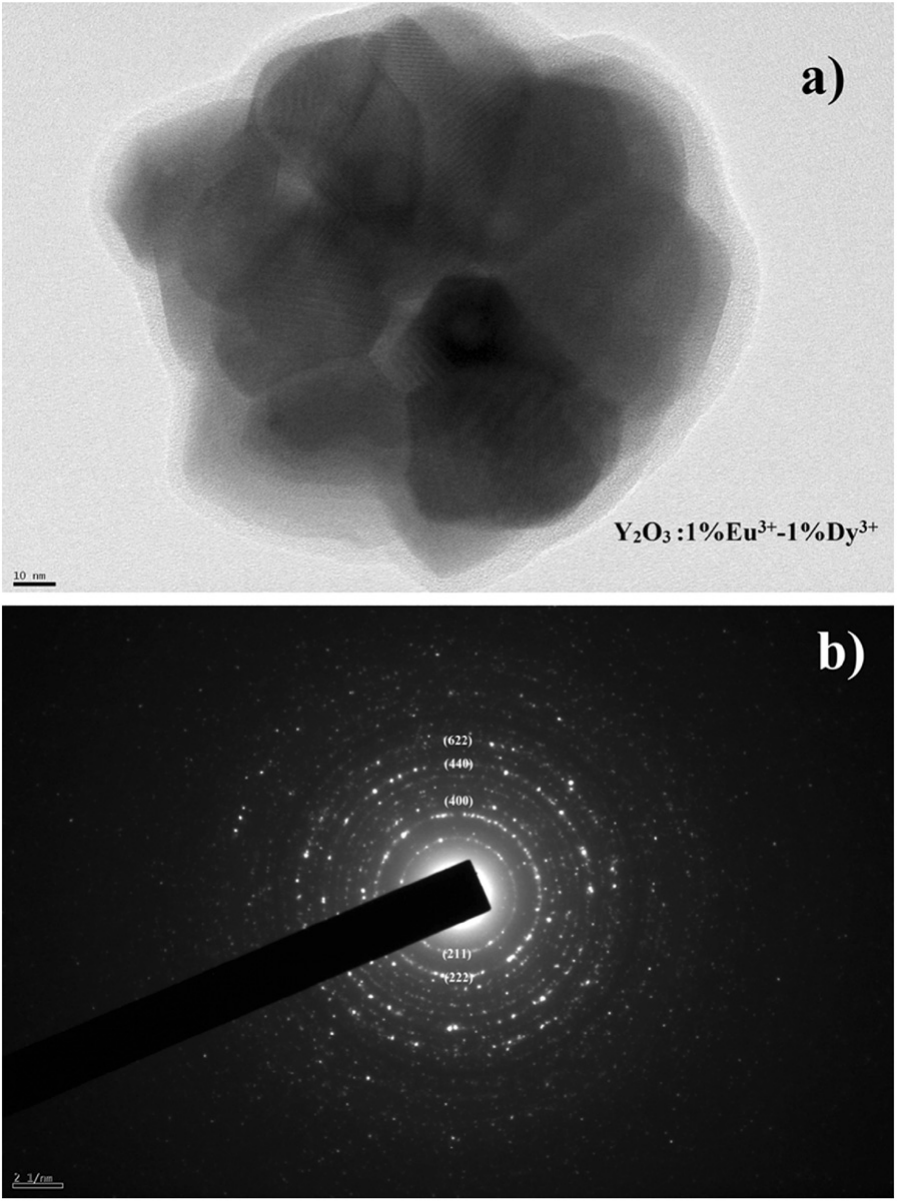
**Single Y**_**2**_**O**_**3**_**:1%Eu**^**3+**^**-1%Dy**^**3+**^**phosphor particle (*T***_**cal.**_**= 1,000°C).** FETEM image (**a**) and corresponding SAED image (**b**).

Figure 3a shows a FETEM image of a single Y_2_O_3_:1%Eu^3+^-1%Dy^3+^ phosphor particle. It is obvious that a single Y_2_O_3_:1%Eu^3+^-1%Dy^3+^ particle has a relatively spherical shape consisting of smaller crystallites (approximately 35 ± 12 nm) associated with each other, which is in good agreement with that calculated from the XRD patterns using the Debye-Scherrer's equation. The lattice fringes in the FETEM image also confirm the high crystallinity of the phosphor product. Figure 3b shows the corresponding selected area electron diffraction (SAED) image of the Y_2_O_3_:1%Eu^3+^-1%Dy^3+^ particle. The clear concentric rings from the inside to outside were indexed directly to the {211}, {222}, {400}, {440}, and {622} planes of cubic Y_2_O_3_, demonstrating the highly crystalline nature of the phosphor particles. 

### Luminescent properties

**Figure 4:**
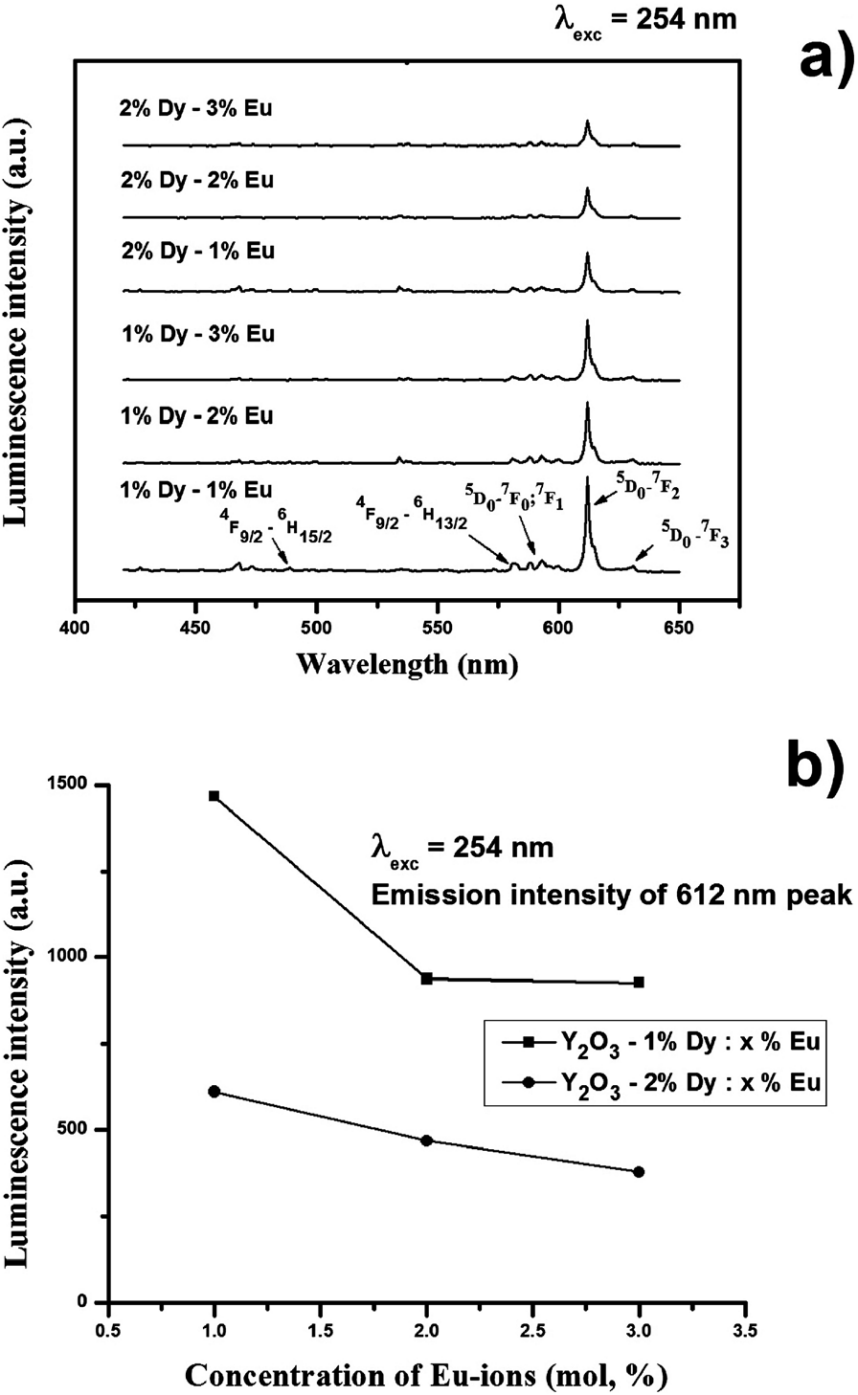
**PL emission spectra and integrated luminescence emission intensity (612-nm peak).** PL emission spectra of Y_2_O_3_ phosphor particles codoped with different Eu^3+^ and Dy^3+^ contents (**a**) and integrated luminescence emission intensity of 612-nm peak as a function of RE ion concentration (**b**) upon constant 254 nm excitation.

As we mention, the luminescence emission of phosphor materials depends strongly on the synthetic route, size of the phosphor materials, and concentration of dopant ions. The particle sizes were similar for all Eu^3+^- and Dy^3+^-codoped Y_2_O_3_ samples which were fabricated under identical conditions. This allows a comparison of the luminescence emission properties of Y_2_O_3_ phosphor particles codoped with different concentrations of Eu^3+^ and Dy^3+^ activators. Figure 4a shows the emission spectra of Y_2_O_3_ phosphor particles codoped with different Eu^3+^ and Dy^3+^ concentrations under ultraviolet (254 nm) irradiation. The emission spectrum showed several main groups of emission lines, which were assigned to the ^5^D_1_ → ^7^F_1_ and ^5^D_0_ → ^7^F_*j*_ (where *j* = 0, 1, 2, and 3) transitions within Eu^3+^[8,12]. Obviously, the emission spectrum is dominated by a red ^5^D_0_ → ^7^F_2_ (612 nm) transition within the Eu^3+^. Figure 4b shows the ^5^D_0_ → ^7^F_2_ (612 nm) transition peak height as a function of the codoped Eu^3+^ and Dy^3+^ concentration under ultraviolet (254 nm) irradiation. According to the recent literature, the best doping value of Eu^3+^ was reported to be approximately 5 mol% in Y_2_O_3_ host material [5,13]. On the other hand, the luminescence intensity of the ^5^D_0_ → ^7^F_2_ (612 nm, hypersensitive to the environment) transition decreased significantly (Figure 4a,b) with increasing codoped Eu^3+^ and Dy^3+^ concentrations in Y_2_O_3_ host. 

**Figure 5:**
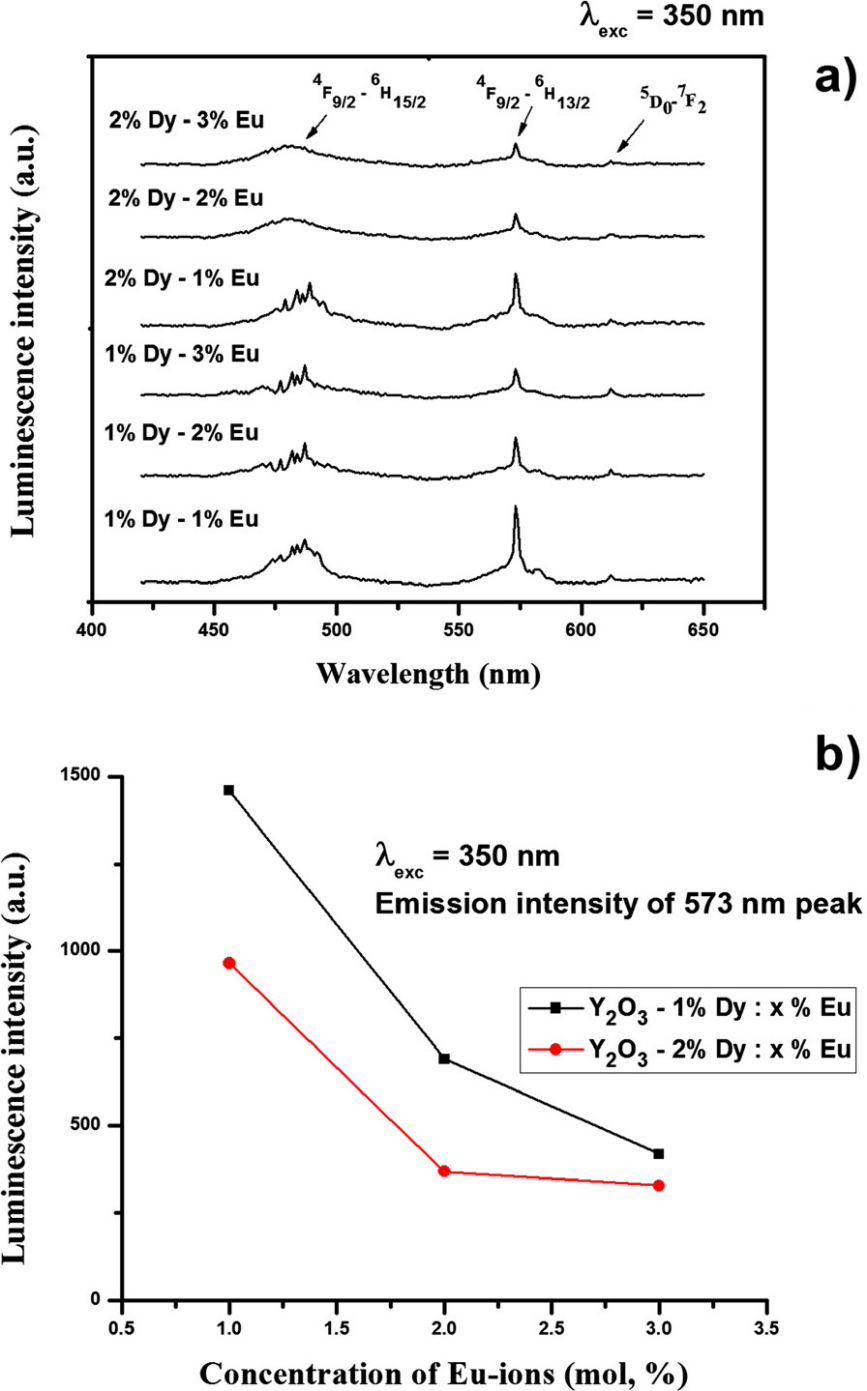
**PL emission spectra and integrated luminescence emission intensity (573-nm peak).** PL emission spectra of Y_2_O_3_ phosphor particles codoped with different Eu^3+^ and Dy^3+^ content (**a**) and integrated luminescence emission intensity of 573-nm peak as a function of RE ions concentration (**b**) upon constant 350-nm excitation.

Figure 5a shows the same samples under 350-nm excitation. In this case, the emission spectrum exhibited luminescence spectra assigned to the characteristic ^4^F_9/2_ → ^6^H_15/2_ (blue region) and ^4^F_9/2_ → ^6^H_13/2_ (greenish-yellow region) transitions within Dy^3+^[11]. The intensity of the Dy^3+^ characteristic emission transitions also decreased strongly with increasing codoped Eu^3+^ and Dy^3+^ concentration, as it shown in Figure 5a,b. The strong quenching behavior at high codoped Eu^3+^ and Dy^3+^ concentrations was related to a cross-relaxation mechanism (nonradiative decay of the two ions to the ground state) within the dopant ions. 

The mean distance between the dopant ions at high concentrations (*R* = 0.62/(*N*)^1/3^, where *N* is the concentration of ions) was much shorter. Therefore, ions can interact through an electric multipolar process leading to energy migration. The dipole-dipole quenching process is inversely proportional to the sixth power of ion-ion separation and, thus, to the square of the dopant concentration [14,15]. In Figures 4a and 5a, except for a decrease of the luminescence intensity, the emission spectrum of Y_2_O_3_ particles codoped with different Eu^3+^ and Dy^3+^ concentrations was similar due to the same *f-f* transitions within the specific RE ion. Therefore, the concentration of codoped RE ions plays an important role and should be strongly considered during the phosphor fabrication process.

**Figure 6:**
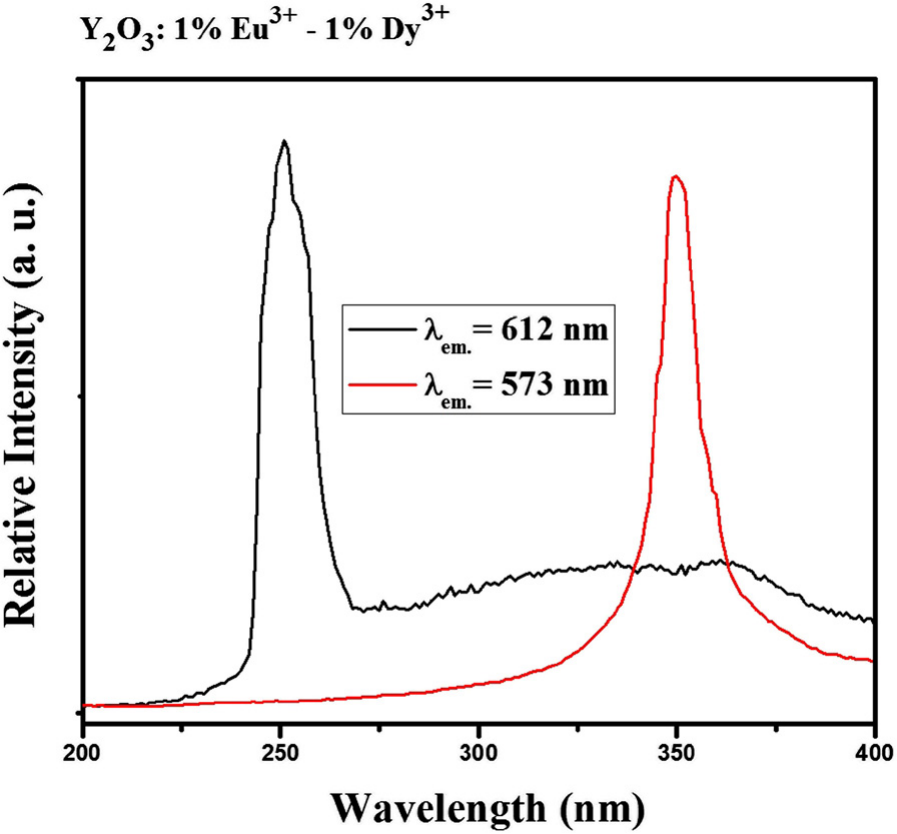
**PL excitation spectra of Y**_**2**_**O**_**3**_**:1%Eu**^**3+**^**-1%Dy**^**3+**^**phosphor particles (*T***_**cal.**_**= 1,000°C).**

**Figure 7:**
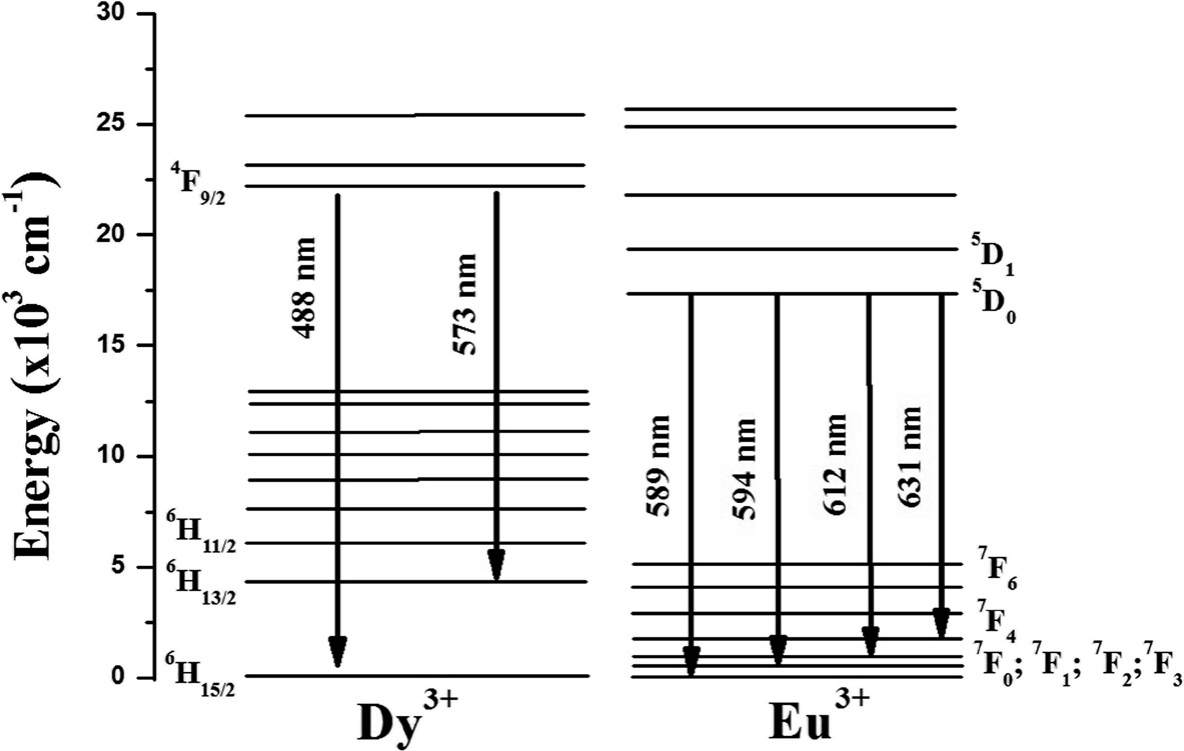
**Schematic diagram illustrating the Dy**^**3+**^**/Eu**^**3+**^**energy level diagram and transitions between the levels.**

**Figure 8:**
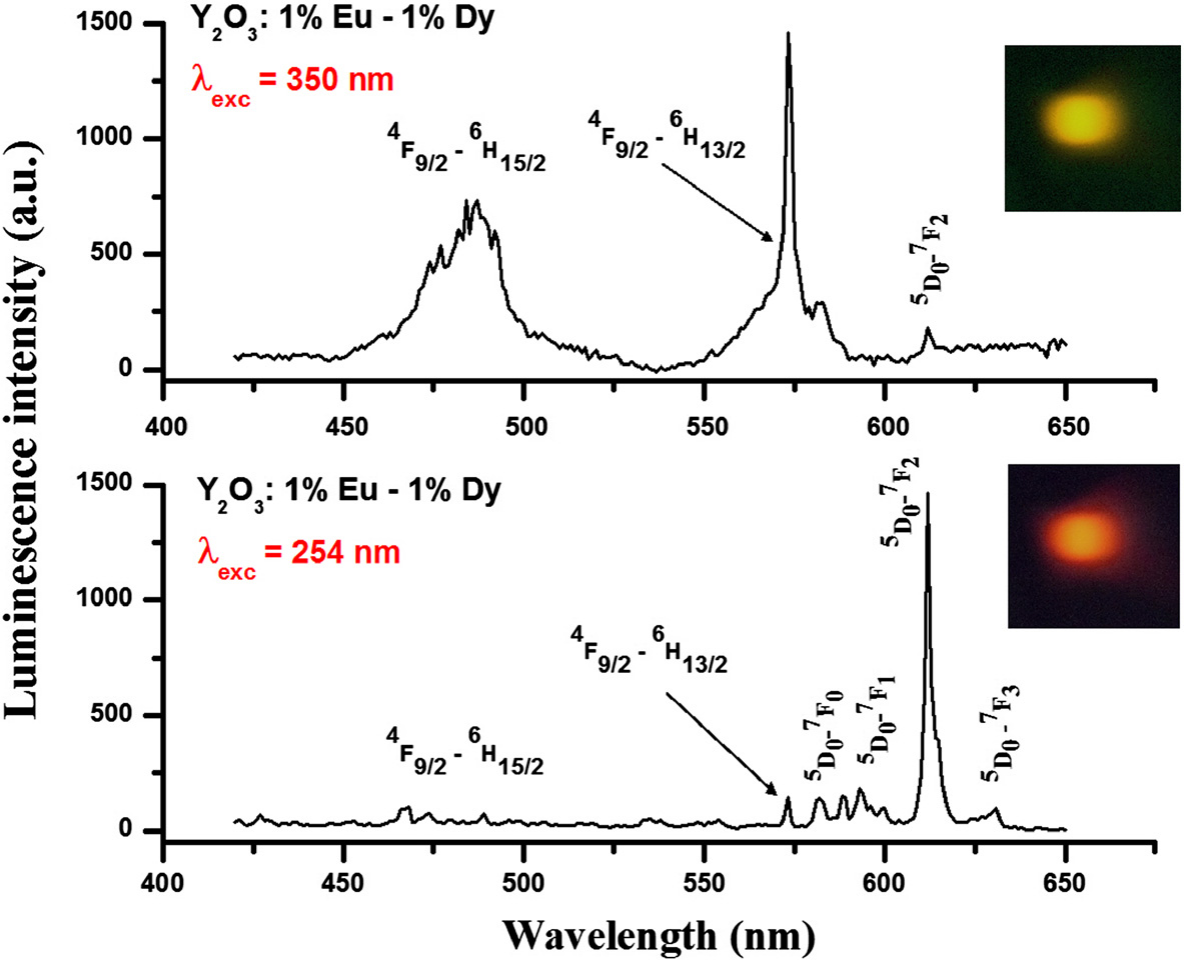
**PL emission spectra of Y**_**2**_**O**_**3**_**:1%Eu**^**3+**^**-1%Dy**^**3+**^**phosphor particles (*λ***_**exc.**_**= 254 and 350 nm, respectively) with assigned transitions.** Inset digital photographs showed the eye-visible red and yellow luminescence emissions.

The excitation spectra of Y_2_O_3_:1% Eu^3+^-1% Dy^3+^ particles are shown in Figure 6. The excitation spectra of Y_2_O_3_:1% Eu^3+^-1% Dy^3+^ (*λ*_em._ = 612 nm) consists of a band extending from approximately 230 to 260 nm, which is related to the charge-transfer band (CTB) of O^2−^-Eu^3+^ bond. Upon excitation into the CTB at 254 nm, the emission spectrum exhibited several emission lines that were assigned to the ^5^D_1_ → ^7^F_1_ and ^5^D_0_ → ^7^F_*j*_ (where *j* = 0, 1, 2, and 3) transitions within Eu^3+^, as shown in the Figure 7. On other hand, the weak signal at 573 nm, which was assigned to ^4^F_9/2_ → ^6^H_13/2_ (hypersensitive transition of Dy^3+^), was also observed, as shown in Figures 7 and 8. Figure 6 also shows the excitation spectra of Y_2_O_3_:1% Eu^3+^-1% Dy^3+^ particles measured in the 200- to 400-nm range by monitoring the yellow emission transition of Dy^3+^ (*λ*_em._ = 573 nm). In this case, when the excitation wavelength was switched to 350 nm, the hypersensitive ^6^P_7/2_ level of Dy^3+^ was excited resonantly, which then quickly relaxes nonradiatively to populate the ^4^F_9/2_ level [11]. Radiative emission occurred from ^4^F_9/2_ to a lower ^6^H_15/2_ and ^6^H_13/2,_ emitting at 488 and 573 nm, respectively, with feeble ET to Eu^3+^. The energy transferred to Eu^3+^ cascades rapidly via nonradiative transitions to the ^5^D_0_ state, from which luminescence associated with Eu^3+^ occurs. Therefore, the feeble signal of the ^5^D_0_ → ^7^F_2_ (612 nm) transition within Eu^3+^ was also detected in Eu^3+^ and Dy^3+^ codoped phosphor particles upon 350-nm excitation. 

Such behavior suggests that there is some energy transfer (ET) occurring between the two codoped ions. On the other hand, the ET observed in Eu^3+^- and Dy^3+^-codoped Y_2_O_3_ was not as strong as that observed in Tb^3+^-Eu^3+^-codoped Y_2_O_3_ phosphor [8]. Therefore, the emission wavelength and color output of the same Y_2_O_3_:1% Eu^3+^-1% Dy^3+^ particles can be adjusted by switching the radiation from 254 to 350 nm. The digital photographs of eye-visible luminescence emissions from Y_2_O_3_:1% Eu^3+^-1% Dy^3+^ particles upon 254 and 350 nm excitations are shown in the Figure 8. It is obvious that the same host material emit red or yellow color, depending on the excitation wavelength.

## Conclusion

In conclusion, Eu^3+^- and Dy^3+^-codoped Y_2_O_3_ submicron spherical particles were synthesized using the urea homogeneous precipitation method. The crystal structure and morphology of synthesized particles were characterized by XRD, FESEM, EDX, and FETEM. The PL spectroscopy was used to examine luminescent properties of Eu^3+^- and Dy^3+^-codoped Y_2_O_3_ particles. PL measurements revealed strong concentration quenching at high-codoped Eu^3+^ and Dy^3+^ concentrations. The luminescence color emission could be controlled by the excitation wavelength and the incorporation of Dy^3+^ and Eu^3+^ at the appropriate concentrations into Y_2_O_3_ structure. Weak energy transfer between the codoped ions was observed. These color-tunable Y_2_O_3_:1%Eu^3+^-1%Dy^3+^ phosphor particles can be used for security printing, solid state illumination, or for optical displays.

## Competing interests

The authors declare that they have no competing interests.

## Authors' contributions

All the specimens used in this study and initial manuscript were prepared by TSA. YHH and HKK added a valuable discussion and coordinated the present study as principal investigators. All authors read and approved the final manuscript.
